# Studying foraging behavior to improve bait sprays application to control *Drosophila suzukii*

**DOI:** 10.1186/s12862-024-02251-0

**Published:** 2024-05-11

**Authors:** K. Escobedo-Quevedo, M. J. Lankheet, I. Pen, M. Trienens, H. H. M. Helsen, B. Wertheim

**Affiliations:** 1 Groningen Institute for Evolutionary Life Sciences, University of Groningen, Groningen, The Netherlands; 2 Wageningen University & Research, Experimental Zoology WIAS, Wageningen, The Netherlands; 3 Wageningen University & Research, Field crops, Randwijk, The Netherlands

**Keywords:** Integrated pest management, Food lures, Spotted wing drosophila, Summer and winter morph, Attraction, Arrestment, Starvation resistance

## Abstract

**Background:**

Foraging behavior in insects is optimised for locating scattered resources in a complex environment. This behavior can be exploited for use in pest control. Inhibition of feeding can protect crops whereas stimulation can increase the uptake of insecticides. For example, the success of a bait spray, depends on either contact or ingestion, and thus on the insect finding it.

**Methods:**

To develop an effective bait spray against the invasive pest, *Drosophila suzukii*, we investigated aspects of foraging behavior that influence the likelihood that the pest interacts with the baits, in summer and winter morphotypes. We video-recorded the flies’ approach behavior towards four stimuli in a two-choice experiment on strawberry leaflets. To determine the most effective bait positioning, we also assessed where on plants the pest naturally forages, using a potted raspberry plant under natural environmental conditions. We also studied starvation resistance at 20 °C and 12 °C for both morphs.

**Results:**

We found that summer morph flies spent similar time on all baits (agar, combi-protec, yeast) whereas winter morphs spent more time on yeast than the other baits. Both morphs showed a preference to feed at the top of our plant’s canopy. Colder temperatures enhanced survival under starvation conditions in both morphs, and mortality was reduced by food treatment.

**Conclusions:**

These findings on feeding behavior support informed decisions on the type and placement of a bait to increase pest control.

**Supplementary Information:**

The online version contains supplementary material available at 10.1186/s12862-024-02251-0.

## Background

For the past decade, agricultural pest control has been evolving, from the indiscriminate use of broad-spectrum chemical pesticides with an unsustainable application schedule, towards a more sustainable strategy based on Integrated Pest Management (IPM). Nevertheless, IPM is not a one-size-fits-all approach; it requires a thorough understanding of pest biology and the environment where it is intended to be used, to determine the optimal timing and application protocol for a pest control strategy [1]. The pest insect’s responses to environmental stimuli and the way that it interacts with its environment are generally manifested in insect behavior [2]. There are many different types of insect behavior, affecting insect foraging (including associative learning and memory, innate preferences, spatial orientation, mobility and flight activity), reproduction (e.g. social groups organization and crowding, aggression, mate selection, mating, reproductive patterns, taking care of offspring), and survival under harsh conditions (e.g. behavioral avoidance of toxins, natural enemies and unfavorable abiotic conditions, including diapause) [3, 4]. Developing knowledge on insect behavior can therefore play an integral part in the optimization of an IPM strategy.

A key aspect of insect behavior is their foraging, i.e. their ability to find a host plant/food resource, the time spent in exploiting the food source, and the feed intake [5]. Insects use cues to locate and assess food sources, including visual and/or olfactory information emitted by the food resource [6, 7]. In pest insects, feeding behavior can be manipulated as part of an IPM control strategy. Feeding can be inhibited by applying deterrents onto valuable crops [8] or it can be stimulated by applying phagostimulants. The latter has been used widely in bait spray approaches against insect pests.

Bait sprays consist of a food lure mixed with a low dose of insecticide that can be applied as a solid/dry or liquid solution [9, 10]. Bait sprays are compatible with IPM because of the potentially lower impact on non-target organisms [11]. The use of a phagostimulant attractant increases the uptake by the pest dramatically lowering the effective dose compared to contact insecticides. When the bait spray is applied on a substrate that is not harvested, such as the leaves in a fruit crop system, this can also reduce the risk of food contamination with insecticides [12]. Bait sprays have proven to be effective in controlling pests such as *Ceratitis capitata*, *Bactrocera oleae*, *Diabrotica virgifera virgifera, *and *Drosophila suzukii* [13–17]. In addition, the quantity of insecticide used, and the number of bait spray applications each year can be reduced [14, 18, 19].

*Drosophila suzukii* is an invasive species that urgently requires the development of effective IPM strategies. It is currently causing agricultural damage to many crops across different continents. This species originates from Asia, but has rapidly colonized almost all of Europe and America. Females have a serrated ovipositor that allows them to lay eggs inside healthy soft fruits. *Drosophila suzukii* larvae develop inside fruits, which potentially transported them to new environments [20, 21]. It also protects them from exposure to insecticides that are sprayed to protect crops [22].

*Drosophila suzukii* adults have a high fecundity and two phenotypic morphotypes that allowing survival traits suited for the seasonal environmental conditions. The summer morph phenotype develops when pupae experience warm temperatures (> 15 °C). These summer morphs are usually predominant from the end of spring and during summer [23, 24]. The winter morph phenotype develops from pupae experiencing low temperatures (< 12 °C) and occurs from autumn to spring in reproductive diapause in northern latitudes [23, 24]. The morphs show differences in several key life history traits and physiology. Summer morph adults emerge with limited glycogen and sugar reserves but they are able to increase their carbohydrate reserves within a day when they have access to a food source [25]. Winter morph adults in response to cold conditions, the most upregulated genes are those involved in metabolic pathways (e.g. glucose metabolism, tricarboxylic acid cycle, and glycogen metabolism) [24]. In addition, winter morph adults storage energy sources which at cold temperatures are needed to survive [26]. Biological control of this invasive pest is so far largely ineffective, and pest control consists mostly of repeated applications of broad-spectrum insecticides [27]. The development of bait sprays could strongly reduce the amount of pesticide used for the management of this insect pest.

The aim of these studies was to investigate the foraging behavior of *D. suzukii* adult females to develop bait spray application protocols tailored to their biology. We aimed to determine (1) *D. suzukii* attraction, arrestment and interaction with baits using video recordings of bait droplets on plant leaves, (2) the position of the baits in the crops that most *D. suzukii* were attracted to, and (3) the impact of starvation on subsequent feeding behavior winter and summer morph adults.

## Materials and methods

### Fly rearing

The strain of *D. suzukii* used was originally initiated from ∼ 300 adults reared from raspberries collected from the Fruit Research Station from Wageningen University & Research in Randwijk, The Netherlands (51.936883 N; 5.705474E), and has been maintained as a large, outbred population since October 2017. *Drosophila suzukii* were cultured in plastic bottles (∼ 140 ml) containing medium food (50 ml) based on water (50 ml), yeast (1.75 g), molasses (1.5 g), dextrose (1.5 g), sugar (0.75 g), cornmeal (0.75 g), agar (0.5 g), soy flour (0.5 g), wheat germ (0.5 g), ethanol (0.5 ml), propionic acid (0.25 g), and tegosept (0.1 g). Female summer and winter morphotype flies were reared in a climate chamber at 20 °C, 16:8 (L: D) and 12 °C, 12:12 (L: D), respectively. Prior to each experiment, female flies were separated from males with a mouth aspirator (without CO_2_ anesthesia) and placed in a plastic bottle with a water moist piece of cotton wool to let them fast for 24 h.

## Approach behavior towards a bait

To identify approach behavior of *D. suzukii* adult females towards a food bait, and their interactions with food baits, we used a two-choice set-up, in which we offered different combinations of baits on strawberry plant leaves on a short stalk (ca. 10 cm tall) in a simple 3-D environment. The females’ behavior was recorded for 3 h after introduction. For each morphotype, eight replicate groups of eight females were recorded for each combination of baits. A single video camera was used for each replication and four cages (with different bait combinations) were recorded at the same time. All the recordings were conducted over a period of 16 different days.

The bioassay was performed in a climate chamber at 20 °C, 16:8 (L: D) in plastic cages of 24 × 12 × 12 cm (Semadeni, Plastic Group, Ostermundigen), with a Plexiglas plate as a lid. Each cage contained a water moistened piece of cotton wool placed in a corner of the cage to prevent desiccation. The video cameras (Panasonic_HC-V, full HD resolution, AVCHD-50p) were used for continuous recording of the behavior in the cages, each camera stationary positioned on a tripod. Depending on the leaf position, the video camera was placed in front of or on the top of the experimental arena, aiming to have a perpendicular view of the leaves.

In each cage a strawberry compound leaf with two opposite leaflets was placed in the middle. The stalk of the leaves was placed in water in a glass cup (100 ml, Duran) which was sealed with Parafilm. A 20 µl droplet of bait was placed on each of the two leaflets. In total, four treatments were tested: (1) water, (2) plain liquid agar (∼ 12%), (3) combi-protec®, a commercial protein-based lure (Vlamings BV), and (4) yeast paste (Mauripan® dry yeast, *Saccharomyces cerevisiae*). Preparations of each treatment are detailed in supplementary information (Table s1). Agar and water were considered negative control treatments, as they had no nutritional value. The combinations offered were: (a) water-agar; (b) combi-protec-agar; (c) yeast-agar; and (d) combi-protec-yeast. In each repetition, a new strawberry leaf was used; the bait position was randomly switched between right and left leaflet and the position of the cages in the climate chamber was rotated.

**Fig. 1:**
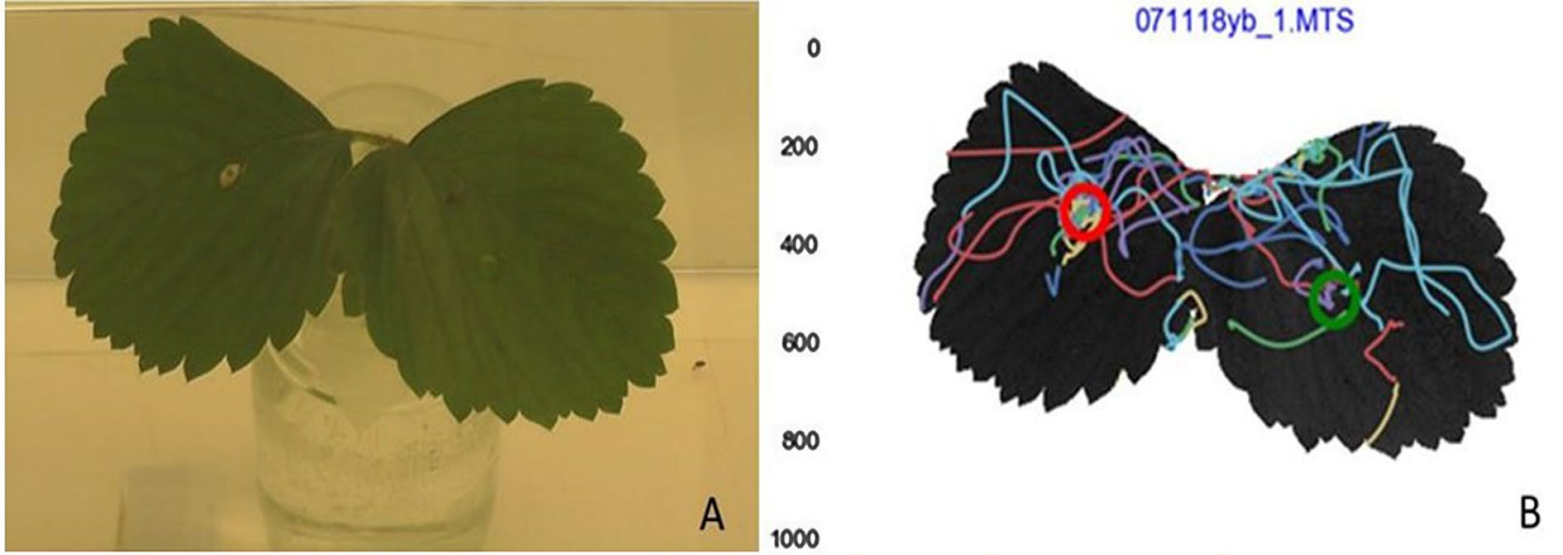
Both pictures show the same strawberry compound leaf with two opposite leaflets. (**A**) An example of the experiment set up with yeast and combi-protec baits (right and left, respectively). (**B**) Tracking software output which shows the flies’ movements on the leaves; each color is a track of a fly’s movement and the red (yeast) and green (combi-protec) circles indicate where the baits were located

In each cage, 8 mated female *D. suzukii* of 15 days old were released in one corner of the cage by gently shaking them out of the bottle. Prior to their release, the flies had been starved for 24 h in a bottle, containing a moist piece of cotton wool. Their foraging behavior towards the baits on the leaves was recorded continuously for 3 h. The 2-D video recording was analyzed at 50 frames per second using in-house (Department of Experimental Zoology, Wageningen University & Research) tracking software (Fig. 1). From the data generated by the software, the measurement we analyzed were (1) the number of visits to each bait, (2) the time spent in immediate contact with the bait, and (3) how long the track was from the first moment flies appeared on the leaflet to the bait location. The number of visits per bait was considered a proxy for the attractiveness of the bait for approach, and more visits to one bait compared to the other was considered as a more attractive bait. The time spent in immediate contact with the bait was considered arrestment on the bait; a measure for their propensity to interact with the baits. Finally, the length of the track from the starting point of a fly’s appearance on the leaflet to the bait location was considered tortuosity, and a proxy for the close-range attractiveness of a bait. Short tracks indicated that flies immediately moved towards the bait once they arrived on the leaflet, whereas longer tracks indicated that the flies walked around before encountering the bait. The same setup was used for summer morph and winter morph flies, except that the winter morph flies were first transferred into a climate chamber at 20 °C, 16:8 (L: D) for 24 h prior to the 24 h starvation to allow for acclimation and to avoid mortality due to temperature shock and relative humidity changes.

### Plant-height feeding preference

This experiment investigated whether *D. suzukii* adult females showed preferences for the heights within a soft-fruit crop plant for feeding. The experiment consisted of a vertical three-choice setting in a potted raspberry (*Rubus idaeus*) plant of approximately 60 cm height. The experiment was done under outdoor conditions in insect cages, at different times of the year. Temperature, humidity and light were recorded every 5 min (HOBO H8 Family data logger, ONSET) to assess the influence of external conditions on plant-height feeding preferences.

**Fig. 2:**
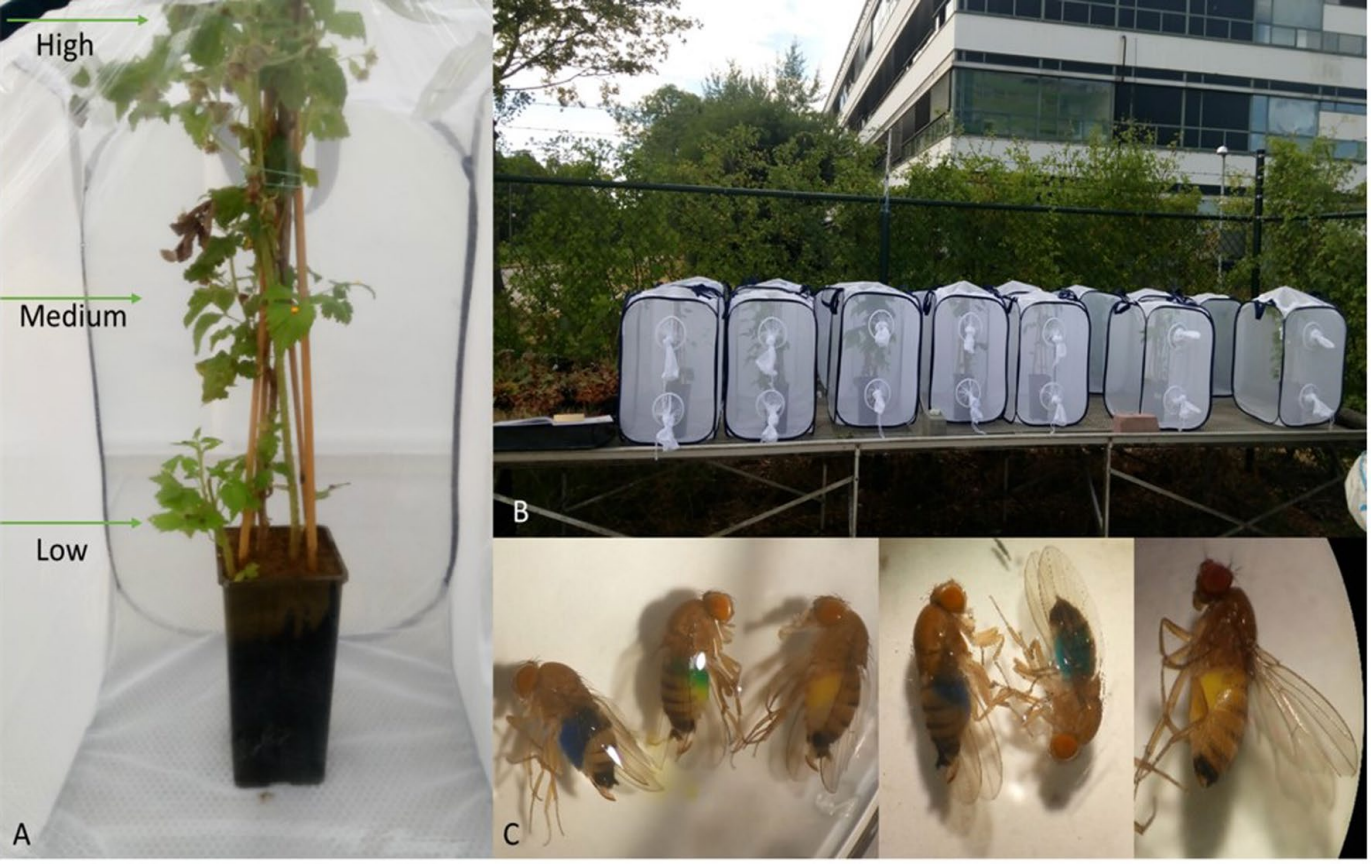
**A** Raspberry plant (∼ 60 cm height) divided into 3 heights. **B** Insect cages with raspberry plants, baits and flies placed under outdoor conditions. **C** Flies with colored abdomen which shows at which height they fed

In this bioassay, 15 replicate insect cages (BugDorm) of 60 × 60 × 90 cm were used in parallel. In each cage a labelled raspberry plant in a pot was introduced and a water moist piece of cotton wool was placed in a corner of the cage to prevent desiccation of the flies. For each plant (∼ 60 cm total height), three height zones were categorized, approximately every 15 cm each: low, medium and high (Fig. 2). In each zone, a randomized leaf was chosen to place a 40 µl droplet of a colored sugar paste as bait food (Dr. Oetker® kleurstoffen) composed of glucose syrup, sugar, carrageenan, colorant, acidity regulators and preservatives. At each height within a single plant, a differently colored bait was offered, to enable us to assess at which height the flies had fed (by the color of their abdomen). The three colorants were randomized for each repetition of the experiment. Within each repetition of the experiments, the three colors were offered at each height within blocks of experimental plants (‘dye swaps’). The plants were divided in three blocks (A, B, C), based on the colors used, e.g., plants in block A contained red droplets at the low height, plants in block B contained red droplets at medium height and plants in block C contained red droplets at the high height.

In each cage, 25 mated, 8–20 days old, female flies were released in one corner of the cage by gently shaking the bottle empty. The flies were allowed to feed for 3 h, after which they were collected with a mouth aspirator and introduced into a test tube with the same label as the plant they were extracted from. Test tubes were placed in liquid nitrogen and flies transferred into a Petri dish where their translucent abdomens were examined with a microscope and recorded for color of ingested food. Flies that showed two different colors in their abdomen (< 13% and < 4% for summer and winter morphs, respectively; no flies were observed that fed on three colors), were discarded from the analysis. In total, eight repetitions of the experiment were done (each with 15 cages), four times for summer morph and four times for winter morph. In each repetition, old used leaves were removed from the raspberry plants and new leaves were chosen to contain the droplet. Repetitions for summer morph were done in July, August, September and October 2018 and for winter morph were done in October, November, December 2018 and March 2019.

### Starvation resistance

In the behavioral experiments we observed reduced, or even a lack of, feeding behavior by winter morph flies, despite the 24 h fasting. We performed an experiment to examine adult female survival and reproduction for different nutritive resources and under the absence of food. The experiments were conducted at two temperatures, with both summer and winter morphs. To produce flies for this experiment, summer morph adult flies were allowed to lay eggs for 24 h at 20 °C, 16:8 (L: D). Adult flies were removed from bottles and bottles with eggs were randomly separated into two groups to be placed under different temperature treatments (either at 20 °C, 16:8 (L: D) or at 12 °C, 12:12 (L: D)) to continue development and generate summer and winter morphs. After their emergence, the flies were placed in new medium bottles. Newly emerged summer morph flies were separated into two groups; one group was kept at 20 °C, 16:8 (L: D) and the other group was transferred to 12 °C, 12:12 (L: D). The same was done with winter morph; a group of bottles was kept at 12 °C, 12:12 (L: D) and the other group was transferred to 20 °C, 16:8 (L: D).

When the flies were 12–15 days old, females were separated from males with a mouth aspirator (without CO_2_ anesthesia) and transferred to vials (Transparent plastic Drosophila tube ps, 25 mm x 95 mm) with different nutritive resources. For each treatment combination (morph, temperature, nutritive treatment), 34 vials were set up, each containing 5 adult female flies.

Female survival and reproduction were compared among two nutritive and one non-nutritive treatments at 12 °C and at 20 °C. The nutritive treatments were (1) sucrose, and (2) fly medium food, and the non-nutritive was (3) water only. For treatments of water and sucrose the vials contained a bed of agar (∼ 6 ml) based on water (6 ml), agar (0.102 g), and nipagin solution (0.102 ml). The water and sucrose treatments were offered on filter paper (1.3 cm x 4 cm); in the medium food vials. Sucrose was prepared as 10 g sucrose with 20 ml water. Filter papers were immersed completely in the solution with metallic tweezers and when removed, touched on one side of the glass to remove excess droplets. For the medium food vials, the plain-agar base was replaced with regular medium food (see above); a same-size filter paper was added to each vial without any additional treatment. This treatment was the control for mortality under optimal food conditions. Every day fly mortality was recorded, and every two days the vials were renewed. All vials that had contained flies were transferred to 20 °C and kept there for a week to be inspected for larval development. After inspection, vials were discarded. This revealed the reproductive activity of the flies under different food conditions.

### Statistical analysis

All statistical analyses were performed in RStudio version 1.4.1103 [28] for Windows.

#### Approach behavior towards a bait

The data was divided into subsets by the combination of baits offered and by summer and winter morph. The combinations offered were: (a) water-agar; (b) combi-protec-agar; (c) yeast-agar; and (d) combi-protec-yeast. Every subset was analyzed individually to compare the behaviors towards two different baits.

To investigate attraction to the bait, we analyzed the number of visits to each bait in a combination using a Wilcoxon paired samples test.

To assess arrestment on a bait, we considered the time spent in immediate contact with the bait. First, we converted the frames to seconds dividing the number of frames by the frame rate (50). We used a general linear model (GLM) with time spent as a response variable, bait as a fixed effect and Tweedie as a family.

To characterize tortuosity, we examined the path travelled from the first appearance of a fly on the leaflet to the bait location. A general linear model (GLM) was used with distance as a response variable, bait as a fixed effect and Tweedie as a family.

For the three different statistical models, we used car: Anova (“car” package [29]) to test significance between baits in the combination offered.

#### Plant-height feeding preference behavior

The response variable in this experiment was categorical (low/medium/high). We used a Cumulative Link Mixed Model with pairwise comparisons and estimated marginal means to compare height preferences, with morph as a fixed effect and date, plant ID (A, B, or C) and bait colors as random effects. We performed estimated marginal means by using emmeans package [30] to contrast the statistics in height preference for the two morphs.

#### Starvation resistance

Mortality was analyzed with Kaplan-Meier (K-M) survival curves and we used log-rank test (LR) to compare the curves between morphs and temperatures. Oviposition (presence/absence larvae) data was analyzed using a Generalized Linear Model (GLM). The dataset was divided into two treatment groups: nutritious (sucrose + food medium) and non-nutritious (water-only). For oviposition data, we also performed emmeans to contrast the statistics in treatment, temperature, and morph.

## Results

### Approach behavior towards a bait

Flies repeatedly approached one or both offered food baits during the three hours of video recording. Most *D. suzukii* females (> 90%) approached a bait by walking up the stalk, whereas some flies first flew and then landed in the vicinity of a bait and then walked towards it. After 1–1.5 h, water and combi-protec evaporated but in the case of combi-protec, flies still visited the place where the droplet was and were able to feed from the dried stain during the entire 3 h of recording.

**Fig. 3:**
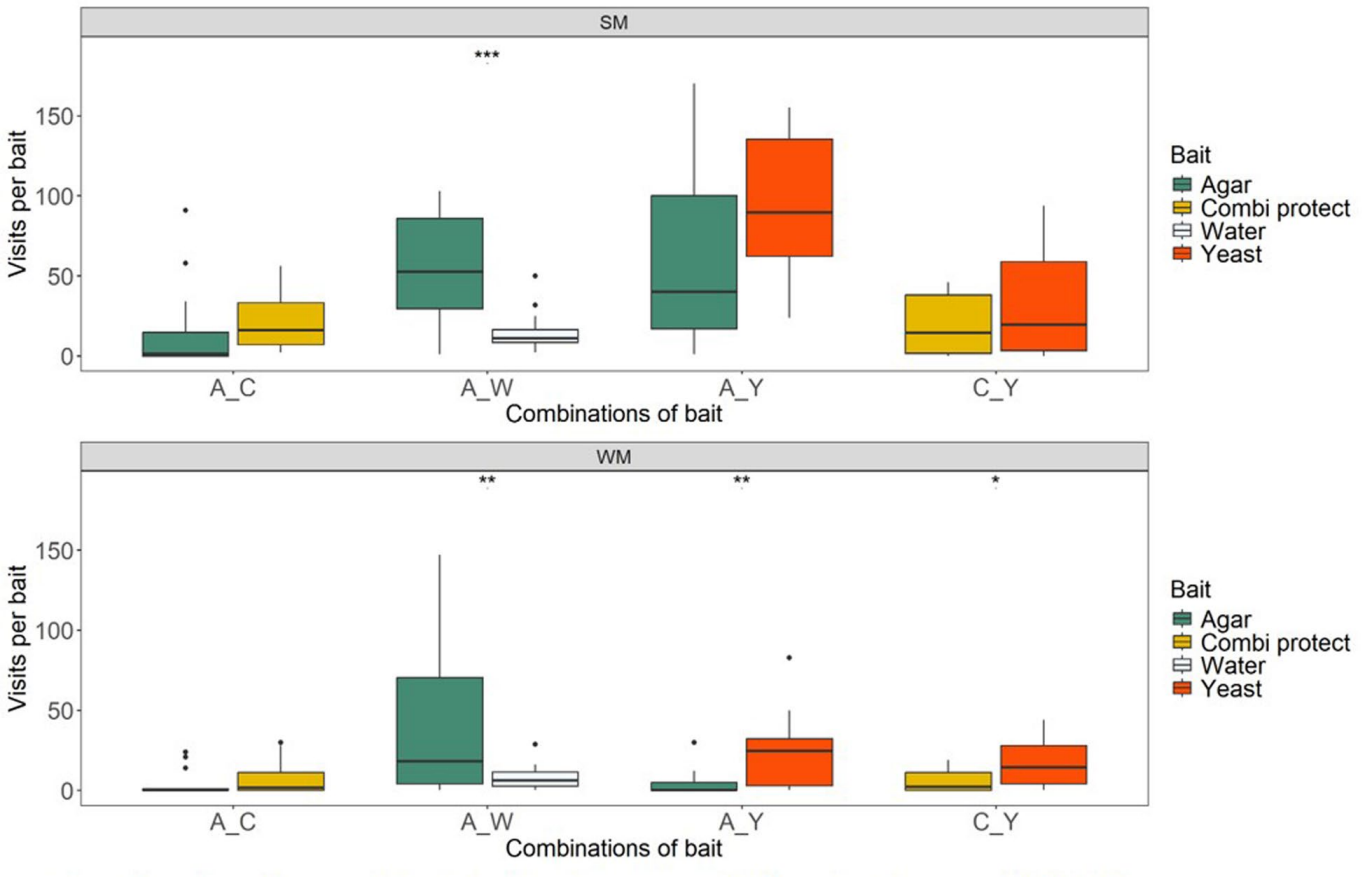
Number of *D. suzukii* visits to baits by summer (**SM**) and winter morph (**WM**) flies when offered a bait in a two-choice set up. In each set up (plastic cage), a combination of baits was offered. Four combinations were used: (1) agar-combi protec (A-C); (2) agar-water (A-W); (3) agar-yeast (A-Y); (4) combi-protec-yeast (C-Y). Horizontal lines represent the median

The number of *D. suzukii* visits to baits depended on the combinations offered (Fig. 3). For the combination of the two control treatments, agar-water, both morphs visited the agar baits more often than the water droplet. The number of visits to the physical presence of the agar was significantly higher (mean for summer morphs, 78 and 15 visits / 3 h for agar and water, respectively; mean for winter morphs, 66 and 8 visits / 3 h for agar and water, respectively) than the number of visits to the location of the (evaporating) water droplet (Wilcoxon paired samples test; *p* = 0.001 and 0.005, summer and winter morph, respectively).

In the agar-combi-protec combination, the number of visits to either bait was relatively low (mean for summer morph, 15 and 20 visits / 3 h for agar and combi-protec, respectively; mean for winter morph, 4 and 7 visits / 3 h for agar and combi-protec, respectively), and there was no preference for visits to either bait, for both morphs (Wilcoxon paired samples test; summer and winter morph, *p* = 0.313 and 0.098, respectively).

When agar and yeast were offered in combination, for summer morphs, the number of visits to agar was highly variable, whereas the number of visits to yeast was relatively high (mean for summer morph, 70 and 93 visits / 3 h for agar and yeast, respectively), However, summer morphs showed no significant preference in this combination (Wilcoxon paired samples test; *p* = 0.313). For *D. suzukii* winter morphs, the number of visits to agar was consistently low (mean = 4), while the visits to yeast were significantly higher (mean = 25; combination agar-yeast, Wilcoxon paired samples test; *p* = 0.002).

When the combination of combi-protec and yeast was provided, the summer morphs did not show a preference (mean for combi-protec and yeast 19 and 34 visits / 3 h, respectively; Wilcoxon paired samples test; *p* = 0.233). However, the winter morphs showed a preference for yeast over combi-protec (mean for combi-protec and yeast 6 and 17 visits / 3 h, respectively; combination combi-protec-yeast, Wilcoxon paired samples test; *p* = 0.030).

**Fig. 4:**
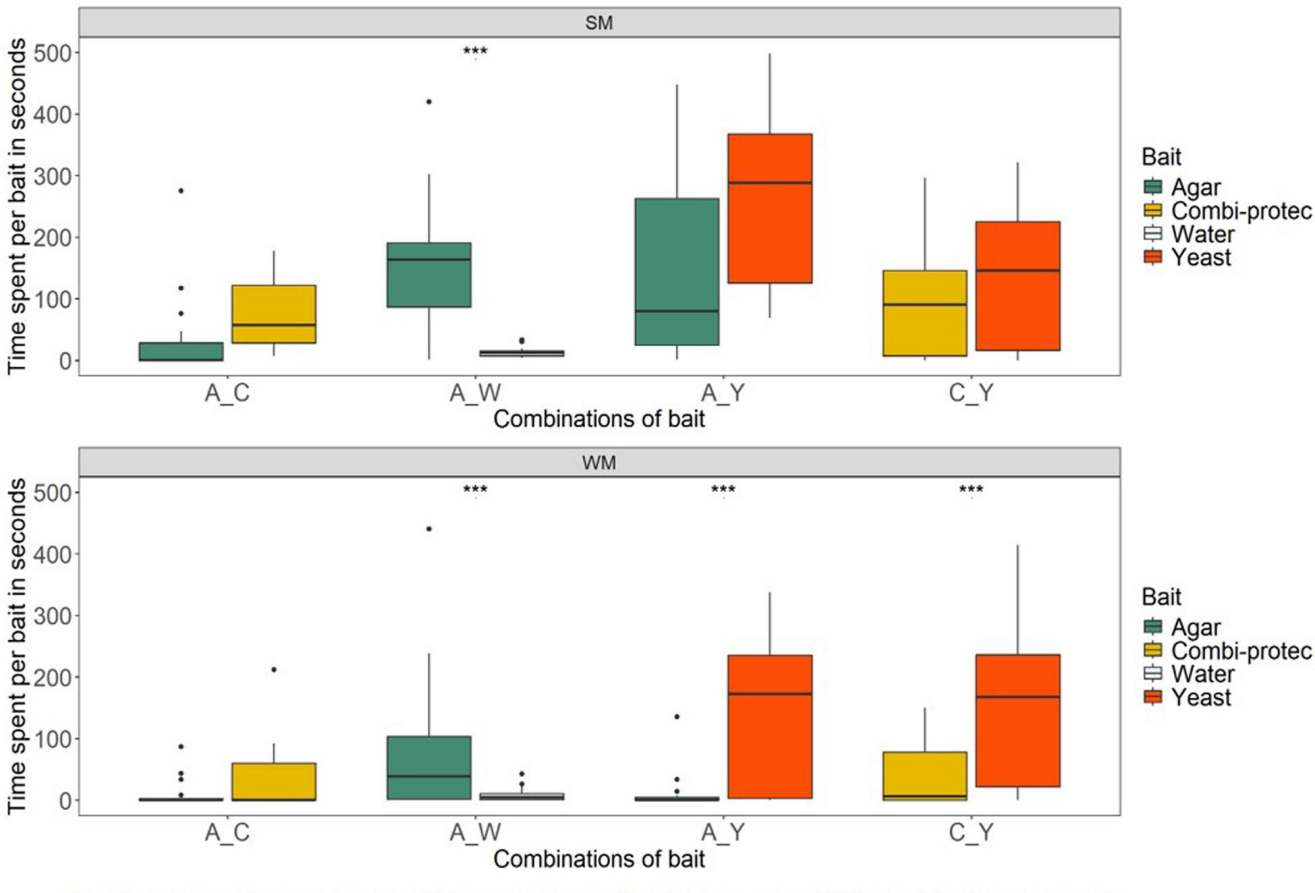
*Drosophila suzukii* time spent on four different baits by summer (**SM**) and winter morph (**WM**) flies. Baits were offered in a two-choice set up. In each test, a combination of baits was offered: (1) agar-combi-protec (A-C); (2) agar-water (A-W); (3) agar-yeast (A-Y); (4) combi-protec-yeast (C-Y). Horizontal lines represent the median

The time that flies spent on the different bait types, offered as two-choice, also depended on the combination provided (Fig. 4). For the combination of the two control treatments, both morphs spent more time on agar than on water (mean for summer morph, 183 and 14 s / 3 h for agar and water, respectively; mean for winter morph, 157 and 9 s / 3 h for agar and water, respectively; combination agar-water, glm; *p* < 0.001 and 0.012, summer and winter morph, respectively), again responding apparently to the physical presence of the non-nutritious baits.

Summer morphs showed a tendency to spend more time on the nutritious baits, combi-protec (mean for agar and combi-protec 35 and 76 s / 3 h, respectively; combination agar-combi-protec, glm; *p* = 0.084), and when presented with yeast (mean for agar and yeast 168 and 267 s / 3 h, respectively; combination agar-yeast, glm; *p* = 0.085). When given two nutritious baits simultaneously, however, the amount of time they spent on combi-protec and yeast was similar (mean for combi-protec 123 and yeast 129 s / 3 h, respectively; combination combi-protec-yeast, glm; *p* = 0.890).

Winter morphs spent more time on yeast, both when the alternative was agar (mean for agar and yeast 12 and 151 s / 3 h, respectively; combination agar-yeast, glm; *p* < 0.001) and when presented with combi-protec as alternative (mean for combi-protec and yeast 38 and 159 s / 3 h, respectively; combination combi-protec-yeast, glm; *p* = 0.002).

When presented with combi-protec and agar, *D. suzukii* winter morph females spent a similar amount of time on the nutritious and non-nutritious bait (mean for agar and combi-protec 11 and 34 s / 3 h, respectively; combination agar-combi-protec, glm; *p* = 0.143).

Distance traveled to find a bait was different between morphs (Supplemental, Fig. s1). The track length for summer morph was longer towards water than to agar (combination agar-water, glm, *p* = 0.007). For the combination agar-combi-protec, the track length of summer morphs was longer towards combi-protec than agar (combination agar-combi-protec, glm; *p* = 0.041). Hence, agar induced a more direct approach of the baits than either the (evaporating) water droplets and the (evaporating) combi-protec droplets. When presented with the agar-yeast combination, the track length towards agar was longer than to yeast (combination agar-yeast, glm; *p* = 0.002). When presented with combi-protec and yeast, no difference in the length of the approach tracks was detected (combination combi-protec-yeast, glm; *p* = 0.682). Winter morphs showed no differences in track length for any combination of bait; agar-water (combination agar-water, glm; *p* = 0.197), agar-combi-protec (combination agar-combi-protec, glm; *p* = 0.75), agar-yeast (combination agar-yeast, glm; *p* = 0.91), or combi- protec-yeast (combination combi-protec-yeast, glm; *p* = 0.923). When we restricted the analyses to the first 1.5 h, when the water and combi-protec droplets had not yet evaporated, we obtained qualitatively very similar results, but with smaller sample sizes.

### Plant-height feeding preference behavior

**Fig. 5:**
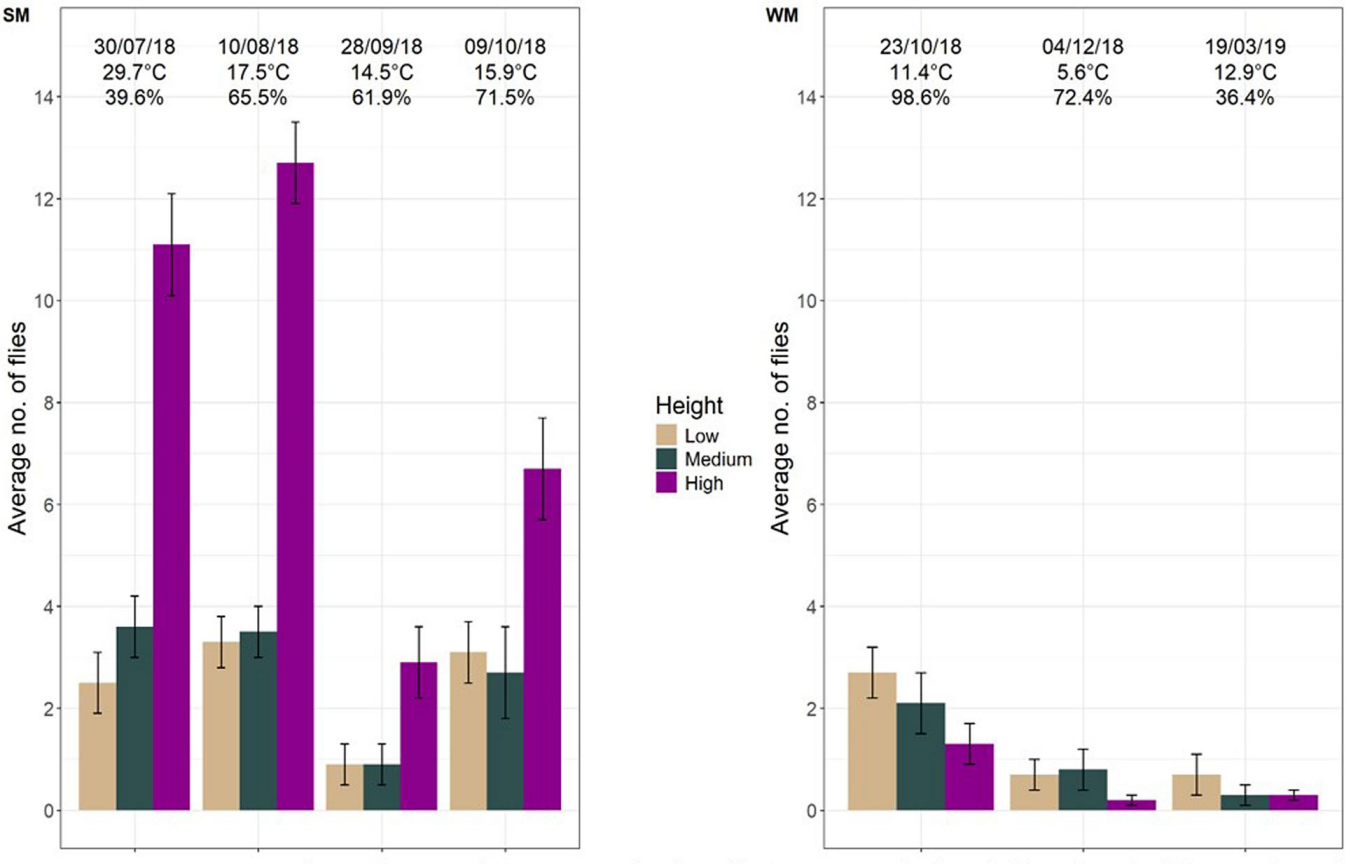
Average number of *D. suzukii* summer (**SM**) and winter morph (**WM**) flies that fed from colored droplets of sugar paste offered at three different heights in a raspberry potted plant in different times of a year. Droplets varied in color with height, allowing identification of feeding location by examining the color of the abdomen of individual flies. The date, temperature, and relative humidity are given at the top of each plot for individual trials

Testing whether *D. suzukii* adult females displayed preferences for the heights within a soft-fruit crop plant for feeding, we found that they mostly fed at the top (∼ 60 cm) of our raspberry plants under semi field conditions compared to 40 cm and 20 cm, regardless of time of the year or weather conditions (Fig. 5). Summer morphs showed a strong preference for feeding at high height (*p* < 0.001 for pairwise comparisons low - high and for medium-high), with 62% of flies feeding at 60 cm and 20% at the medium and low height (Supplemental Fig. s2). The remaining flies did not feed. Winter morph flies did not show a clear preference, with 45% feeding at the highest point and 20% and 35% for medium and low height, respectively (*p* = 0.021, pairwise comparisons for medium-high), and no preference between high-low plant positions (*p* = 0.523, pairwise comparisons).

The two morphs differed in their feeding height preference (clmm; *p* < 0.001). The number of flies that fed during assays was substantially higher for summer morph than winter morph (clmm; *p* < 0.001; Supplemental, Fig. s2).

Environmental conditions (e.g. date, temperature, relative humidity) were also measured. The feeding height preference behavior was affected by date (clmm; *p* < 0.001) but not by the separate environmental conditions measured in this study. It is important to note that the environmental conditions were not manipulated independently. There were differences in feeding behavior among dates, but this cannot be attributed to any specific environmental conditions, as they co-vary.

### Starvation resistance

**Fig. 6:**
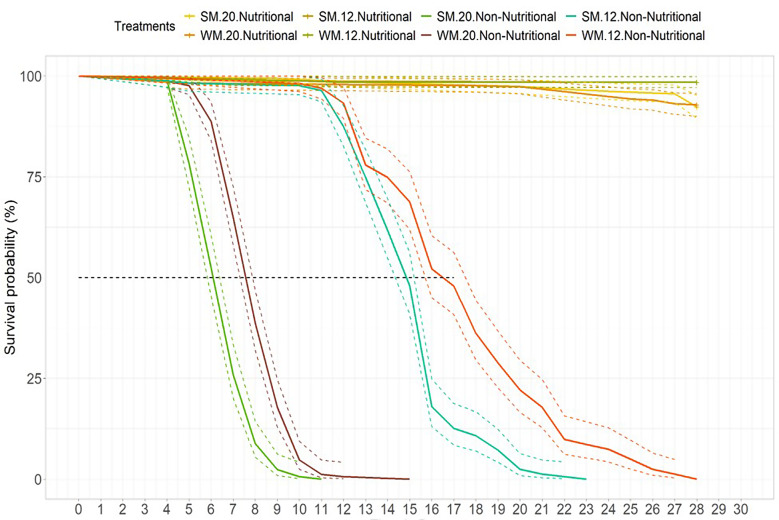
Survival rate in days of *D. suzukii* summer (**SM**) and winter (**WM**) morph flies under nutritional and non-nutritional treatments at two different temperatures. The nutritional treatment consisted of two different treatment groups: sucrose and food medium; and the non-nutritional treatment was water-only

From our previous experiments, we observed few winter morph flies being active or feeding; due to this lack of engagement, we examined adult female survival and reproduction under nutritious and non-nutritious (absence of food) treatments, at 20 °C and 12 °C for summer and winter morphs. Under nutritious treatments, only < 1% of the flies died during the 28 days of the experiment. Under non-nutritious treatments at 20 °C, 50% mortality in summer and winter morph was reached by day 6 and 7, respectively. Under non-nutritious and colder conditions (at 12 °C), 50% of summer and winter morphs were dead by day 15 and 17, respectively (Fig. 6).

In general, the mortality of the two morphs under starvation conditions was mainly affected by temperature (K-M; *p* = 0.001). Colder temperatures (12 °C) enhanced survival compared to warmer temperatures (20 °C) for both morphs. At 20 °C, we observed mortality from four days onwards, whereas at 12 °C this happened after 12 days. At both temperatures, winter morphs endured starvation on average for slightly longer than summer morphs, (LR; *p* = 0.006 at 20 °C and LR; *p* = 0.03 at 12 °C; Supplemental, Fig. s3).

**Fig. 7:**
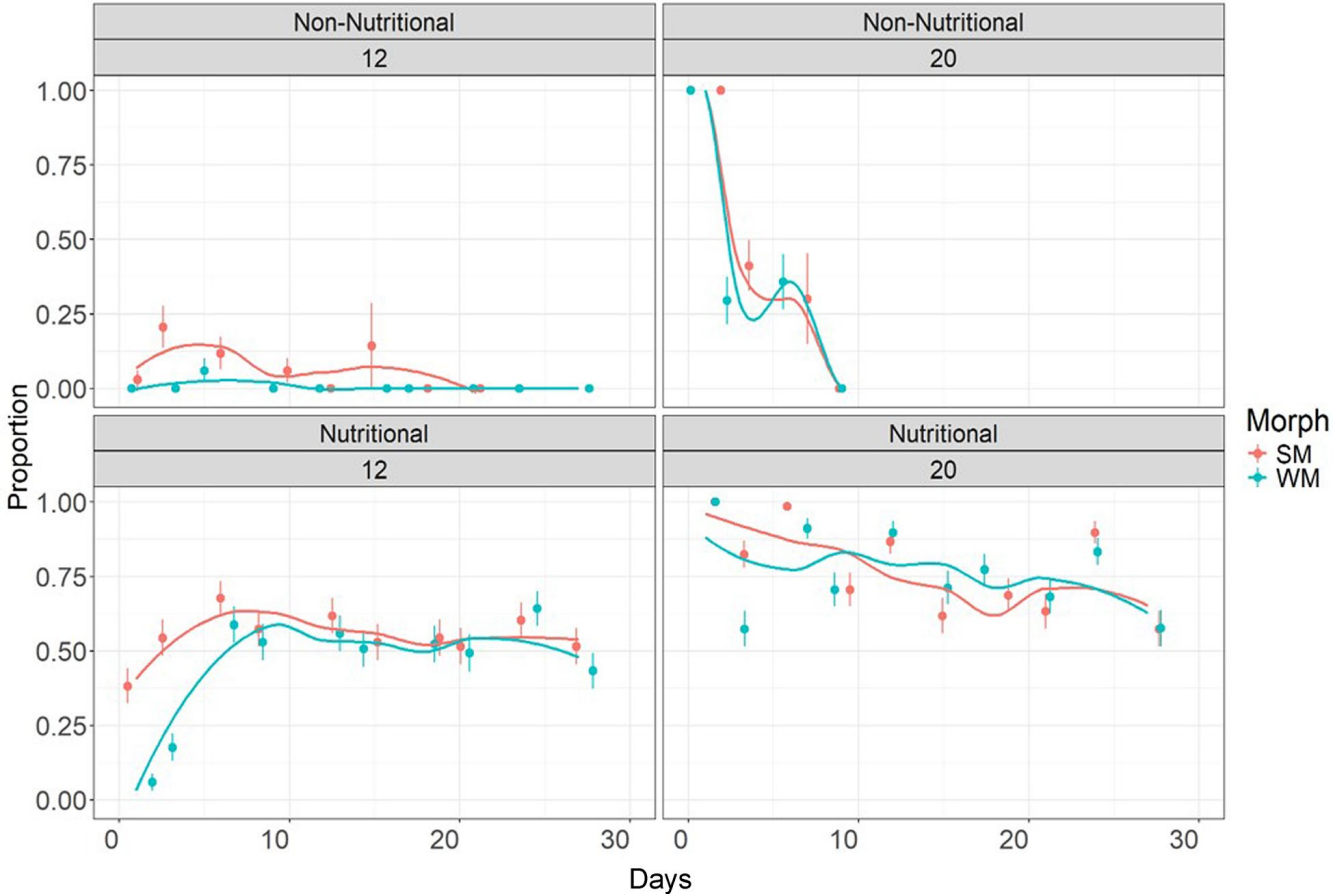
Proportion of *D. suzukii* summer (**SM**) and winter (**WM**) morph flies that oviposited under nutritious and non-nutritious treatments at two different temperatures: 12 °C and 20 °C across 30 days. The nutritional treatment consisted of two different treatment groups: sucrose and food medium; and the non-nutritional treatment was water-only

Reproduction was mostly eliminated under non-nutritious conditions, whereas it was maintained throughout the 28-day period under nutritious conditions (Fig. 7). In non-nutritious conditions under 12 °C, the proportion of winter morph flies that oviposited was close to zero, whereas a small proportion of summer morph flies continued to lay (a very low quantity of) eggs until 20 days. At 20 °C non-nutritional conditions, the proportion of flies from both morphs laid a high number of eggs on the first day, while in later days we saw a sharp decrease in oviposition to no eggs after 9 days.

The proportion of females that oviposited was not constant over time under the two different nutritious treatments and temperatures (Fig. 7; glm; Day: Treatment: Temperature, *p* < 0.001).

Under nutritious conditions, in the first three days, at 12 °C, we observed fewer winter morph vials with eggs and larvae (18%) than in those of summer morph (54%). At around 10 days, we saw a similar number of vials with presence of eggs and larvae in both morphs (53% and 57% for winter and summer morph, respectively). Under nutritious conditions, at 20 °C, we counted more summer morph vials with larvae present (82%) than for winter morphs (57%) during the first 3 days, but after 12 days, more winter morph vials had larvae present (> 70%) than those from summer morphs (62%; *p* < 0.001, pairwise comparisons for Morph-Treatment-Temperature) up to day 27, where there was a reduction in reproduction over time in both morphs (< 60%). Under low temperature (12 °C) and nutritious conditions, there were fewer vials with eggs and larvae (< 65%) than at warm temperature (20 °C, > 65%). At low temperature and nutritious conditions, the number of vials with larvae increased over the first 10 days. This trend was stronger for winter morphs (glm; Morph: Treatment: Temperature, *p* = 0.026), from 6% of vials with larvae to 59% (Fig. 7).

## Discussion

We studied the behavior of summer and winter morph *D. suzukii* adult females in the laboratory towards bait droplets in order to inform future bait spray application. In general, the commercial product (combi-protec) and the yeast (*Saccharomyces cerevisiae*), nutritious baits, increased the number of visits by the adult flies to leaves.

The experiments performed in this manuscript showed that the type of bait and its position within a plant influenced the feeding behavior of *D. suzukii* females. Additionally, we observed behavioral differences between summer and winter morphs towards the baits and in terms of starvation resistance.

During the experiment where we video recorded foraging behavior towards a bait solution, we observed that most of the flies approached the bait by walking and we did not observe high flight mobility. Flies that flew first landed on the outer part of the leaf and then approached the bait by walking. The low flight mobility may be due to the relatively small size of the plastic containers, which could have prevented the flies from flying. Alternatively, it is a possibility that the flies did not fly because they were deprived of food for 24 h prior to the experiments. A previous report indicated that *D. suzukii* females starved for 24–72 h fly less compared to those that have been fed [31]. Even when flies were sated, it was rare that they flew under experimental conditions [31]. The lack of flying behavior may be relevant to take into consideration for application of bait sprays. It could suggest that bait sprays are best applied near the ripening crops, where the flies can easily approach it through walking. Additionally, flying behavior towards a bait under field conditions remains yet to be determined.

The behavior towards a bait depended on the combinations offered to the flies. In general, all baits were visited, some more than others. Yeast baits attracted a high number of visits and flies were arrested for the longest time on these baits. Yeast was selected as bait, because previous research had indicated that many *Drosophila* species, including *D. suzukii*, showed an attraction to yeast volatiles [32, 33]. Hence, using a lure including yeast would make it relatively easy to find over longer ranges. The number of visits to combi-protec were similar compared to the agar control treatment, in line with a low attractiveness. For the time spent, there was a tendency for summer morphs to interact more with the combi-protec bait than with the agar. During the course of the behavioral observations, the droplet of combi-protec dried up, but flies returned to the place where the bait was positioned, and remained there for a period of time: in the combination agar-combi-protec, summer morphs spent an average of 82 s per visit, and winter morphs spent an average of 32 s; in the combination of combi-protec-yeast, summer morphs spent an average of 52 s and winter morphs spent an average of 19 s. When combi-protec and yeast were offered as alternatives, winter morph flies had a higher number of visits and more time spent on the yeast baits, but this was not the case for the summer morph flies.

We had considered agar a control treatment, as a non-attractive (inodorous) and non-nutritious bait (starvation experiment where flies did not survive when kept in vials with an agar bed as an only source of food); nevertheless, flies still visited agar and when it was offered in combination with water, both morphs spent more time on agar. Summer morphs tended to remain at agar for longer periods than winter morphs. Summer morph flies did not discriminate in number of visits among combi-protec, agar, and yeast combinations and once encountered, summer morphs spent similar time near any type of bait; winter morph flies did show stronger preferences in their behavior by visiting and spending more time on yeast when offered (combination agar-yeast and combi-protec-yeast). It is unclear why the agar bait engaged the flies. Possibly, it provided a visual or odorous stimulus that the flies explored, or it may have created a slightly improved micro-climate that arrested the flies. Irrespectively, the observation that the flies interacted to a similar degree with the agar bait could suggest that even a neutral bait may be sufficient to manipulate pests into interacting with a bait spray. However, whether the use of a non-attractive bait such as agar may be effective in a bait spray protocol remains to be determined. Furthermore, flies can encounter other types of food besides the baits that are being offered, and attractive baits may have a higher chance of being competitive in engaging the flies in such situations.

The distances traveled towards baits were considered a proxy for short-range attractiveness, with shorter tracks indicating a more direct approach (or less tortuosity). Summer morph flies indeed walked shorter distances to approach yeast bait compared to agar, in line with the expectation of yeast being a highly attractive bait. However, when either combi-protec or water was offered, which evaporated over the course of the experiment, summer morph approached the alternative agar bait through shorter tracks. This perhaps suggests that visual stimuli are important for finding baits. Winter morph flies did not follow this pattern; their approach routes were similar (short) distances to any bait. This partially reflects that summer morphs were much more active than winter morphs, and spent more time walking on the leaflets. The winter morph flies seemed less explorative, and walked shorter distances overall. This may also affect the chances that the winter morph flies would randomly encounter a bait.

Our feeding-height preference experiment revealed that for both morphs of *D. suzukii*, the height of bait application mattered for how much the flies fed from it. Females of both morphs primarily fed at the top of the plants (∼ 60 cm). Possibly, *D. suzukii* preferred the microclimate offered at the highest height of our plant; indeed microclimates are found to vary in different plant zones (e.g., upside or underside of a leaf, canopy) and may differ at distances less than 40 cm [34]. It might also be that the upper plant of our potted raspberry plants had more or bigger leaves than the lower part. It is also known that *D. suzukii* distribution varies depending on the crops; for instance, *D. suzukii* infestation was influenced by canopy density in cranberries, but not in blueberries [34]. Our plants were relatively small compared to commercial raspberry crops, and tested in isolation from other plants; this makes it difficult to generalize our findings to other settings. It is important to remark, though, that our findings suggest that the height position of the bait spray could play a role in its effectiveness. It has also been shown that height played a role in trap efficacy in field conditions, where traps suspended at 1 to 1.5 m above the soil captured the highest rate of *D. suzukii* compared to traps located at ground level and 2 m above the soil [35].

Our results in the starvation assay corresponded with the previous findings that *D. suzukii* female adults under warm temperatures survived up to 6–7 days with water only [36]. Longevity in mated *D. suzukii* female adults was mainly affected by feeding status and temperature; under *ad lib* food conditions, female adult summer morph average lifespan ranged from 80 days under warm conditions (22 °C) [37] to less than 7 days under cold conditions (0 °C); whereas the lifespan for winter morphs, was 109 and 7 days at 21.5° and 0 °C, respectively [38]. For cooler temperatures of around 15 °C, it has been observed that the median female lifespan was approximately 200 days for summer morphs and approximately 150 days for winter morphs [39]. Winter morph flies endured starvation conditions slightly better than summer morph flies in our experiment. It has already been established that summer and winter morphs differ in physiology and life history traits [40]. This also could cause different nutritional requirements and metabolism, which seem to allow winter morphs to endure non-favorable conditions for longer. *Drosophila suzukii* reared at 10 °C has a different metabolic profile compared to 25 °C by showing an accumulation of cryoprotectants such as polyols and amino acids [41]. Also, in our experiment, there was a large difference in survival under starvation conditions between warm and cold temperatures; cold temperatures prolonged starvation resistance for both morphs, surviving up to 8 days more than under warm conditions. One of many explanations could be that flies were able to reduce energy use and suppress metabolism for short periods under cold temperatures to survive [42].

The proportion of flies that laid eggs was significantly different for non-nutritious and nutritious treatments, and also showed striking differences between temperatures and between morphs. At 12 °C under starvation conditions, a larger proportion of the summer morph flies continued to lay eggs, whereas the proportion of winter morph flies that oviposited was close to zero. Also, under nutritious conditions at 12 °C, a larger proportion of summer morphs initially oviposited, although this gradually converged to similar proportions after 10 days at which stage flies were already 16 days old. This may suggest that not all winter morph flies have fully matured ovaries at 16 days old [43] and most of the flies became reproductively active after 26 days old at 12 °C. Alternatively, the winter morph flies were in reproductive diapause (reproduction arrestment until favorable conditions are present). Even after returning to warm conditions, the production of the first offspring of overwintered winter morphs captured from field took more days than the flies that did not go through a diapause period [44]. At 20 °C and under nutritious conditions, similar proportions of winter morph and summer morphs laid eggs. Under starvation conditions at 20 °C, both morphs very rapidly ceased ovipositing. Overall, in the starvation experiment, there were slight differences between morphs but still these seem insufficient to fully explain the lack of engagement by winter morphs shown during the experiments. An alternative explanation is that winter morphs need more than 24 h to adapt to a new temperature change.

The observed differences among winter morphs and summer morphs may have implications for the development of the protocol on bait spray application. In laboratory conditions, we observed that long-range attractive baits were preferred by winter morphs, whereas for summer morphs an odorless lure, such as agar, can still cause a feeding behavior effect on the flies. The lack of feeding behavior by winter morphs in this study might indicate that the baits that we tested might not be suitable to control winter morph flies. Moreover, it might suggest that a different strategy than bait sprays is needed to control winter morph flies. Both morphs have not only physiological differences and dietary needs, but also seemingly different preferences towards a bait. It has been suggested that winter morph flies during winter might be exploiting food sources associated with moisture on decomposing vegetation [45], whereas summer morph flies might feed from plant leaves and fruit pulp [36], fruit/tree sap, extrafloral and floral nectar [25]. Additionally, phagostimulatory effects of specific species of yeast, such as *Candida zemplininia*, varied between both morphs, with winter morph females showing a greater attraction compared to summer morph females [46]. Moreover, distinct reactions to chemical compounds have been observed between summer and winter morphs. For example, geosmin and methyl salicylate repelled summer morphs but not winter morphs under laboratory conditions [47]. It might be that the baits used in this study were not phagostimulatory enough for winter morph flies and further research is needed to generate more knowledge on winter morph flies lure preferences.

## Conclusion

Bait sprays have been used to control different pests. We found the bait type and height on the plant that the bait was applied impacted the interaction of *D. suzukii* females with the bait. It is important to consider that control strategies might differ depending on the morph to control, as we found differences in their foraging behavior and starvation resistance. We studied this in controlled and simplified environmental settings, to obtain detailed insight into aspects of *D. suzukii* foraging behavior. To extrapolate our findings to field conditions, field research in agricultural settings is needed. We believe that tailoring the application protocols to the biology and foraging behavior of the pest can indeed contribute to enhanced efficacy and efficiency of bait sprays.

### Electronic supplementary material

Below is the link to the electronic supplementary material.

Supplementary Material 1

## Data Availability

Data and scripts are made publicly available through DataverseNL. https://dataverse.nl/privateurl.xhtml?token=546c4edf-3bc2-4043-8a4dbdfada6789a5.
