# Supplementary material for: Studying foraging behavior to improve bait sprays application to control Drosophila suzukii
Source: BMC Ecol Evol. 2024 May 11;24:60. doi: 10.1186/s12862-024-02251-0 (PMC11088012; doi:10.1186/s12862-024-02251-0)
Supplement: Supplementary file 1 — Supplementary Material 1 [file 12862_2024_2251_MOESM1_ESM.docx]

**Supplementary information**

**Table s1** Preparation of solutions offered as food baits to *D. suz*ukii in a strawberry compound leaf for the approach behavior experiment.

| Stimuli | Preparation |
| --- | --- |
| Agar (Caldic ingredients) | 14 gr + 100 ml H_2_O |
| Combi-protec (protein-based attractant; https://combi-protec.com/) | 14 gr + 150 ml H_2_O |
| Yeast (Mauripan® dry yeast) | 24 gr + 70ml H_2_O |


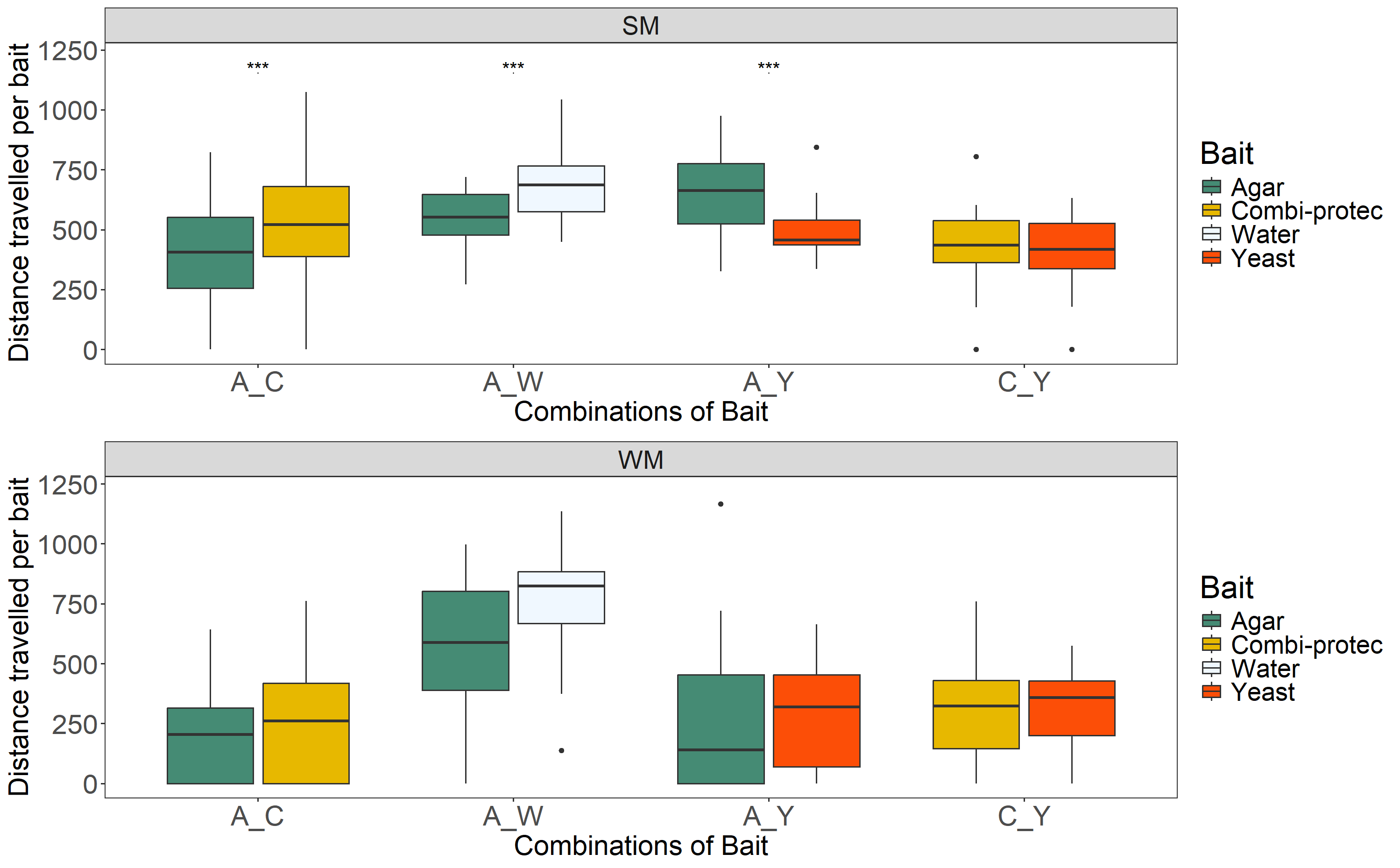


**Fig. s1** Distance travelled by summer (**SM**) and winter morph (**WM**) flies towards a bait offered in a combination. Distance was considered from the first point of appearance of the fly on the leaf to the position of the bait.


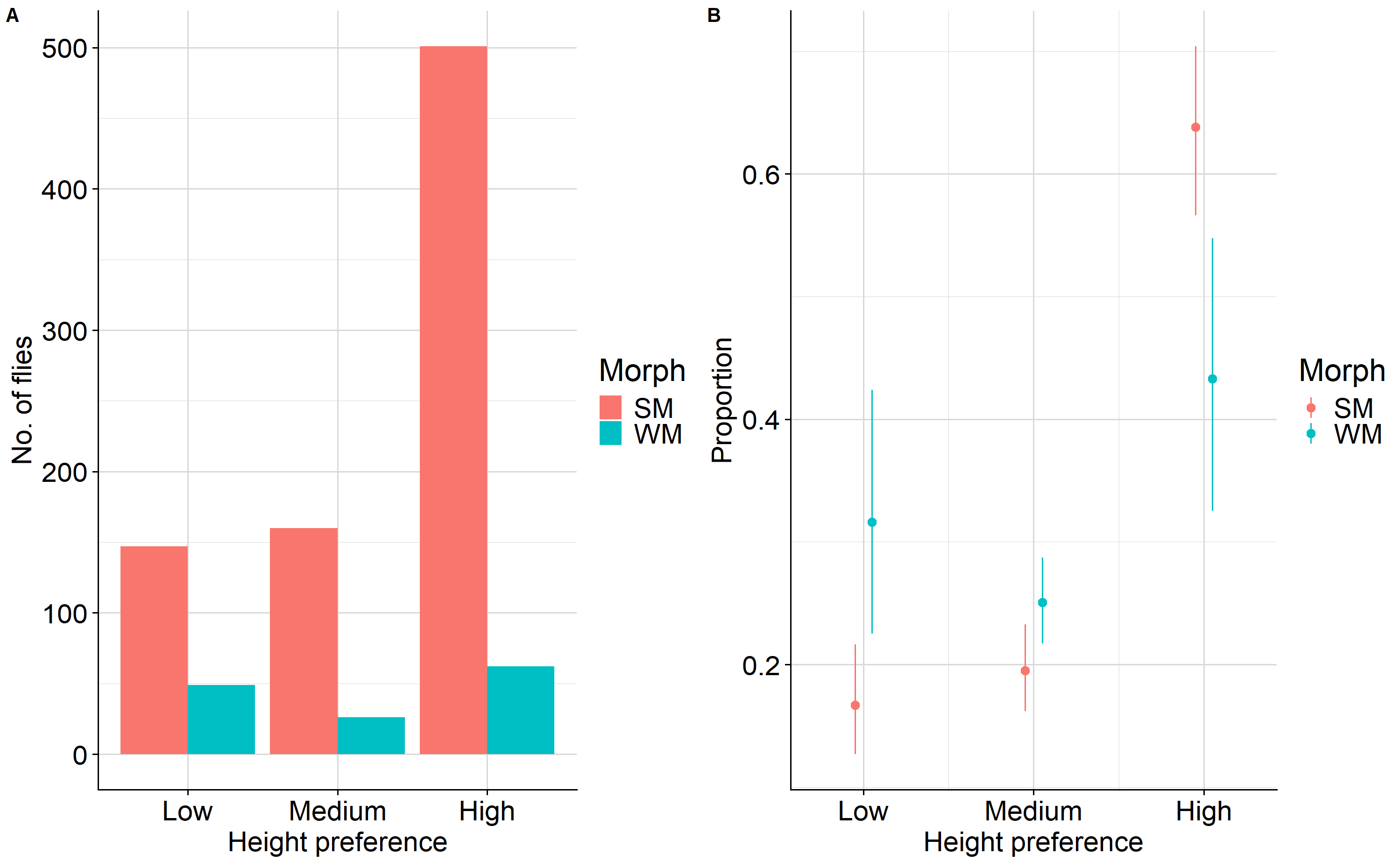


**Fig. s2 A.** Total number of summer (**SM**) and winter morph (**WM**) flies that fed from colored droplets offered in three different heights in a raspberry potted plant. In each height, a different color of droplet was offered to separate flies by height preference depending on their colored abdomen. **B.** Percentage of total summer and winter morph flies that chose among low, medium and high height based on the colored abdomen.


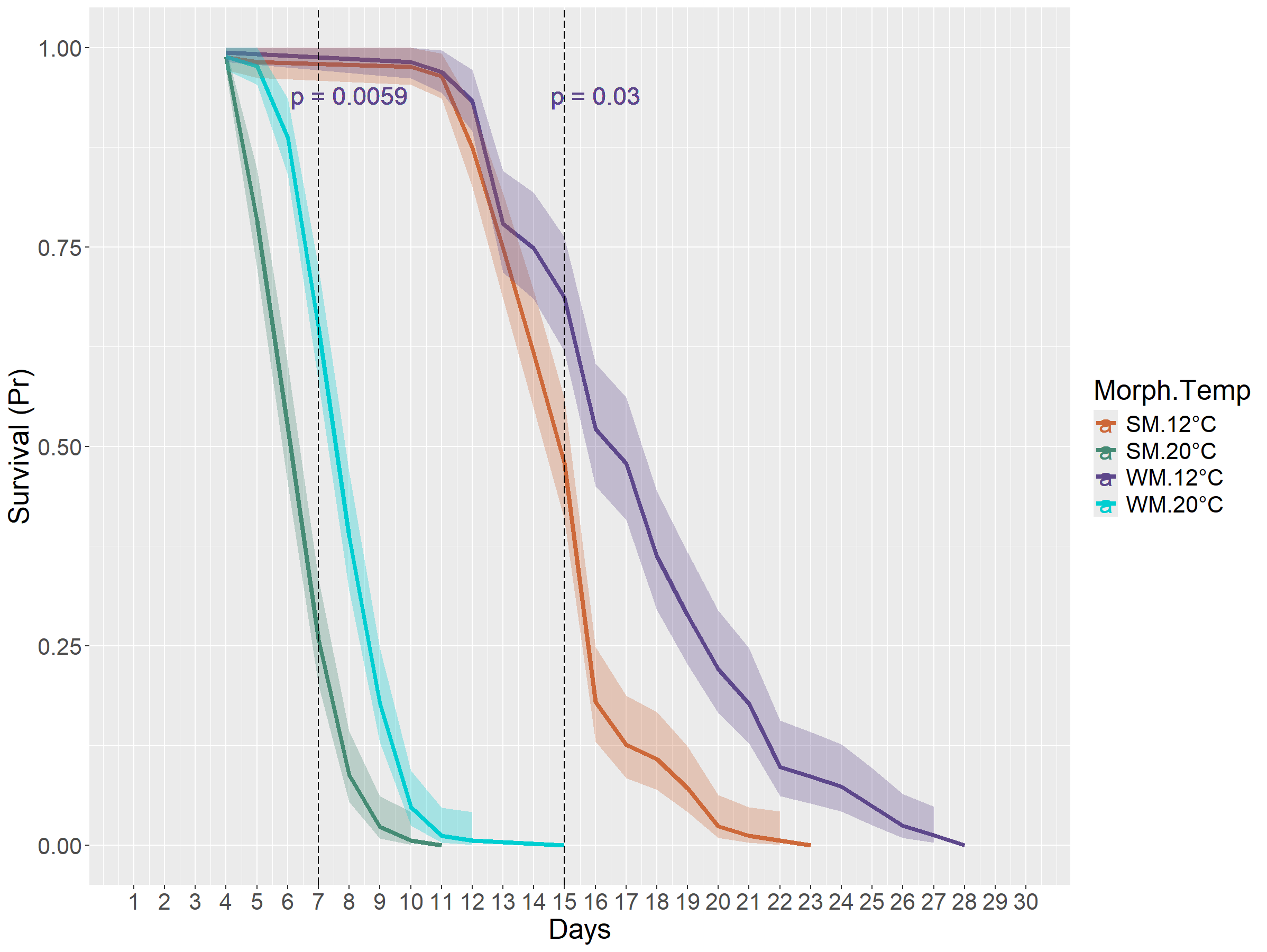


**Fig. s3** Survival of *D. suzukii* summer (**SM**) and winter morph (**WM**) females under non-nutritious conditions (absence of food) under 12°C and 20°C. Mortality started at day 4 onwards and there is a statistical (LR) significance between morphs at day 7 and 15.
